# Association of Cerebral Blood Flow With Longitudinal Changes in Cerebral Microstructural Integrity in the Coronary Artery Risk Development in Young Adults (CARDIA) Study

**DOI:** 10.1001/jamanetworkopen.2022.31189

**Published:** 2022-09-12

**Authors:** Mustapha Bouhrara, Curtis Triebswetter, Matthew Kiely, Murat Bilgel, Sudipto Dolui, Guray Erus, Osorio Meirelles, Nick R. Bryan, John A. Detre, Lenore J. Launer

**Affiliations:** 1 Laboratory of Clinical Investigation, National Institute on Aging, National Institutes of Health, Baltimore, Maryland; 2 Laboratory of Behavioral Neuroscience, National Institute on Aging, National Institutes of Health, Baltimore, Maryland; 3 Department of Radiology, University of Pennsylvania, Philadelphia; 4 Center for Biomedical Image Computing and Analytics, University of Pennsylvania, Philadelphia; 5 Laboratory of Epidemiology and Population Science, National Institute on Aging, National Institutes of Health, Baltimore, Maryland; 6 Department of Diagnostic Medicine, University of Texas, Austin; 7 Department of Neurology, University of Pennsylvania, Philadelphia

## Abstract

**Importance:**

Decreased cerebral tissue integrity and cerebral blood flow (CBF) are features of neurodegenerative diseases. Brain tissue maintenance is an energy-demanding process, making it particularly sensitive to hypoperfusion. However, little is known about the association between blood flow and brain microstructural integrity, including in normative aging.

**Objective:**

To assess associations between CBF and changes in cerebral tissue integrity in white matter and gray matter brain regions.

**Design, Setting, and Participants:**

In this longitudinal cohort study, magnetic resonance imaging was performed on 732 healthy adults from the Coronary Artery Risk Development in Young Adults (CARDIA) study, a prospective longitudinal study (baseline age of 18-30 years) that examined participants up to 8 times during 30 years (1985-1986 to 2015-2016). Cerebral blood flow was measured at baseline (year 25 of the CARDIA study), and changes in diffusion tensor indices of fractional anisotropy (FA) and mean diffusivity (MD), measures of microstructural tissue integrity, were measured at both baseline and after approximately 5 years of follow-up (year 30). Analyses were conducted from November 5, 2020, to January 29, 2022.

**Main Outcomes and Measures:**

Automated algorithms and linear mixed-effects statistical models were used to evaluate the associations between CBF at baseline and changes in FA or MD.

**Results:**

After exclusion of participants with missing or low-quality data, 654 at baseline (342 women; mean [SD] age, 50.3 [3.5] years) and 433 at follow-up (230 women; mean [SD] age, 55.1 [3.5] years) were scanned for CBF or FA and MD imaging. In the baseline cohort, 247 participants were Black (37.8%) and 394 were White (60.2%); in the follow-up cohort, 156 were Black (36.0%) and 277 were White (64.0%). Cross-sectionally, FA and MD were associated with CBF in most regions evaluated, with lower CBF values associated with lower FA or higher MD values, including the frontal white matter lobes (for CBF and MD: mean [SE] β = −1.4 [0.5] × 10^−6^; for CBF and FA: mean [SE] β = 2.9 [1.0] × 10^−4^) and the parietal white matter lobes (for CBF and MD: mean [SE] β = −2.4 [0.6] × 10^−6^; for CBF and FA: mean [SE] β = 4.4 [1.1] × 10^−4^). Lower CBF values at baseline were also significantly associated with steeper regional decreases in FA or increases in MD in most brain regions investigated, including the frontal (for CBF and MD: mean [SE] β = −1.1 [0.6] × 10^−6^; for CBF and FA: mean [SE] β = 2.9 [1.0] × 10^−4^) and parietal lobes (for CBF and MD: mean [SE] β = −1.5 [0.7] × 10^−6^; for CBF and FA: mean [SE] β = 4.4 [1.1] × 10^−4^).

**Conclusions and Relevance:**

Results of this longitudinal cohort study of the association between CBF and diffusion tensor imaging metrics suggest that blood flow may be significantly associated with brain tissue microstructure. This work may lay the foundation for investigations to clarify the nature of early brain damage in neurodegeneration. Such studies may lead to new neuroimaging biomarkers of brain microstructure and function for disease progression.

## Key Points

**Question:**

Is cerebral blood supply associated with cerebral tissue integrity?

**Findings:**

In this cohort study of 732 healthy adults, at 5-year follow-up, significant associations between lower regional cerebral blood flow and decreased integrity of brain tissue were observed in critical brain regions. The association was most pronounced in white matter tissue.

**Meaning:**

This finding suggests that cerebral blood flow might be an early risk factor of future change in cerebral tissue integrity among healthy adults.

## Teaser

This cohort study investigates associations between cerebral blood flow and decreased integrity of brain tissue in healthy adults at 5-year follow-up.

## Introduction

Degradations in brain white matter (WM) and gray matter (GM) and reduction of cerebral blood flow (CBF) are features of several neurodegenerative diseases, including Alzheimer disease and small-vessel ischemic disease.^1,2,3,4,5,6,7^ In particular, vascular dysfunction has been implicated as a potential underlying mechanism for age-related brain parenchymal deterioration.^8,9,10,11,12^ Indeed, brain tissue maintenance is an energy-intensive process, making cerebral tissue homeostasis particularly susceptible to hypoperfusion and concomitant hypoxia or hypoglycemia.^13^ Growing evidence indicates that cerebral microstructural tissue changes, including breakdown of the myelin sheaths, axonal damage, and dendritic or synaptic loss, may be early phenomena in neurodegeneration. Consistent with these observations, there is evidence of a direct association between reduced brain perfusion and neurodegeneration.^14^ Only a few magnetic resonance imaging (MRI)–based studies have investigated the potential association between WM or GM integrity and CBF status. Existing studies have been primarily limited to the context of leukoaraiosis and Parkinson disease^14,15,16^ and indicate that deficits in CBF may trigger brain tissue deterioration. However, the association between blood supply to the brain and cerebral tissue integrity in cognitively unimpaired individuals has received little attention. Examining the extent of this potential association is sine qua non for understanding interactions between CBF and tissue integrity in impaired individuals.

Diffusion tensor imaging (DTI) is an MRI technique sensitive to the underlying microarchitectural status of the brain parenchyma and the degree and direction of water molecule mobility^17^ and has been extensively used to study brain maturation and degeneration.^18,19,20^ DTI provides measures of fractional anisotropy (FA), mean diffusivity (MD), axial diffusivity, and radial diffusivity that are proxies of neuronal status, including axonal, myelin, dendritic, and synaptic integrity. Studies have shown that reduced FA concomitant with an increase in radial diffusivity is associated with demyelination,^21^ whereas an increase in axial diffusivity is associated with axonal damage or death.^22^ Cerebral blood flow can be quantified noninvasively with arterial spin–labeled perfusion MRI.^23^ Arterial spin labeling (ASL) uses arterial blood water protons labeled by radiofrequency pulses as an endogenous tracer^24^ and derives CBF from the differences between images acquired with and without this labeling.^24^ Mainly owing to its noninvasive nature and the widespread availability in most MRI machines, ASL has become an indispensable CBF imaging technique in investigations of the brain.^24,25,26,27,28^

Using DTI and ASL, Chen et al^8^ found that lower cortical CBF was associated with lower WM integrity in a cross-sectional study conducted with a cohort of 105 adult participants. This finding was interpreted as an indicator of myelin loss. Furthermore, Giezendanner and colleagues^29^ found that subcortical CBF was associated with the integrity of GM regions and WM tracts in a cohort of 47 participants. Recently, in a study conducted with a cohort of 67 participants and using an advanced MRI method to quantify myelin content,^30,31^ evidence was provided of an association between lower myelin content and lower CBF.^32^ All these findings suggest that the blood supply to the brain may be an important determinant of cerebral tissue health and a potential therapeutic target of neurodegeneration. However, although the aforementioned studies provide pivotal evidence about the intimate association between brain perfusion and cerebral tissue status, they have 2 main limitations: the limited sample sizes investigated and the studies’ cross-sectional nature. Additional longitudinal studies are required.

The Coronary Artery Risk Development in Young Adults (CARDIA) study includes a large and longitudinal cohort of well-characterized adults and examines the development and determinants of clinical and subclinical cardiovascular disease and their risk factors^33^; it also provides the opportunity to extend observations on the association between CBF and DTI indices to a large and longitudinal cohort of adult participants. The CARDIA study began in 1985 with participants aged 18 to 30 years at baseline and examined participants up to 8 times during 30 years (1985-1986 to 2015-2016). A large subset of 732 CARDIA participants underwent an MRI protocol for CBF mapping and DTI at 25 years (Y25) after baseline, with most of them (n = 488) undergoing follow-up at 30 years (Y30) after baseline.^25^ From this subset, Launer and colleagues^25^ showed significant associations between GM CBF or WM FA and various cardiovascular risk factors. These results indicate that variations in brain microstructure are associated with age and various modifiable cardiovascular indicators and motivate the investigation of the potential direct association between CBF deficits and cerebral microstructural damage.

We used CARDIA study data to examine the association between CBF measured at baseline (Y25) and changes in longitudinal DTI indices (namely, FA and MD from Y25 to Y30). We hypothesized that lower CBF at baseline would be associated with lower tissue integrity at baseline. Most important, lower CBF at baseline would be associated with greater rates of decline in microstructural integrity (ie, a greater longitudinal decrease in FA or a greater increase in MD). Our main goal was to provide new insights into the functional coupling between brain blood supply and brain microstructure, thus showing an association between neurovascular physiology and cerebral tissue integrity.

## Methods

### Study Cohort

Participant data for this cohort study were obtained from the CARDIA study.^33^ All participants provided written informed consent at each examination, and the study was approved annually by the institutional review boards from all coordinating centers.^25^ Further details about the CARDIA study and the study cohort can be found in the eMethods in the Supplement. In this study, we used the MRI data of CBF acquired at Y25 of follow-up, which represents the first point of CBF MRI acquisition in the CARDIA study, and of the DTI MRI data acquired at Y25 and Y30 of follow-up. This study followed the Strengthening the Reporting of Observational Studies in Epidemiology (STROBE) reporting guideline.

### MRI Data Acquisition

For each participant, the MRI protocol consisted of sagittal, T1-weighted, 3-dimensional, magnetization-prepared, rapid-gradient echo data acquired with a repetition time of 1900 milliseconds, an echo time of 2.9 milliseconds, an inversion time of 900 milliseconds, a flip angle of 9°, a bandwidth of 170 Hz/pixel, a voxel size of 1 × 1 × 1 mm^3^, a 256 × 256 matrix, 176 slices, and 2 generalized autocalibrating partial parallel acquisitions. Arterial spin–labeled data were acquired using pseudocontinuous labeling with a labeling time of 1.48 seconds and a postlabeling delay of 1.5 seconds at 9 cm below the center of the imaging volume. The images were obtained with non–background-suppressed, 2-dimensional, gradient-echo, echoplanar imaging with a repetition time of 4000 milliseconds, an echo time of 11 milliseconds, a voxel size of 3.4 × 3.4 × 5 mm^3^, a 64 × 64 matrix, a flip angle of 90°, 2 generalized autocalibrating partial parallel acquisitions, a bandwidth of 3004 Hz/pixel, an echo spacing of 0.44 milliseconds, and an echoplanar imaging factor of 64. Twenty slices with a distance factor of 20% were acquired from inferior to superior sequentially. Forty label or control pairs were acquired for signal averaging. Finally, the DTI protocol for FA and MD mapping consisted of diffusion-weighted images acquired with echoplanar imaging; a repetition time of 7.3 seconds; an echo time of 84 milliseconds; 2 *b* values of 0 and 1000 seconds/mm^2^, with the latter encoded in 32 directions; and an acquisition voxel size of 2.19 × 2.19 × 2.2 mm^3^.^25^ All acquisition parameters were identical between scanners and sites (eMethods in the Supplement).

### Image Processing, Parameter Mapping, and Selection of Regions of Interest

An initial quality-control protocol was conducted to identify motion artifacts or other quality issues. Images failing this test were subsequently discarded from our analysis. After the quality-control protocol, the pseudocontinuous ASL^24^ and DTI^17,34^ MRI data sets were processed with automated pipelines for the calculation of the corresponding CBF or FA and MD maps, respectively. Ten regions of interest (ROIs) were defined corresponding to whole-brain GM or WM and frontal, parietal, temporal, and occipital lobe WM or GM. Within each WM or GM ROI, the mean FA and MD values were calculated, whereas the mean CBF values were calculated in the GM ROIs only. Our main analysis was restricted to GM CBF owing to the high sensitivity to noise of CBF values derived from ASL in WM ROIs, in addition to the fact that the pseudocontinuous ASL protocol used here was optimized for the determination of GM CBF.^24^ Further details can be found in the eMethods in the Supplement.

### Statistical Analysis

Analyses were conducted from November 5, 2020, to January 29, 2022. We investigated the association between GM CBF calculated over the whole-brain and regional DTI indices (ie, FA and MD) calculated in each defined GM or WM ROI; we will refer to this analysis as whole-brain analysis. We also investigated the associations between regional GM CBF and GM or WM DTI indices calculated in each ROI; we will refer to this analysis as ROI analysis. Linear mixed-effects model regression analysis was applied using the mean DTI indices of FA or MD within each ROI as the dependent variable and the mean CBF value at baseline, age at the MRI scan, time at and from the MRI scan, race (self-reported as Black or White), sex, and time × CBF as the independent variables. To facilitate interpretation, regional CBF values were mean centered. A random effect was included for intercept per participant. The explicit regression model is given by the following equation^35^:

FA*_ij_* or MD*_ij_* = β_0_ + β_sex_ × sex*_i_* + β_race_ × race*_i_* + β_age_ × age*_i_* + β_time_ × time*_ij_* 

+ β_CBF_ × CBF*_i_* + β_time × CBF_ × CBF*_i_* × time*_ij_* + ε*_ij_* + *b_i_*,

where FA*_ij_* and MD*_ij_* (millimeters squared per second) are, respectively, the FA and MD measures of participant *i* at time *j* (ie, Y25 and Y30), age is the age (in years) at baseline (Y25), CBF*_i_* is the CBF at baseline (milliliters per 100 g/min), time*_ij_* is the time at 0 or approximately 5 years from baseline of participant *i* at time *j*, *b_i_* ~ *N*(0, σ^2^*_b_*) is the random intercept for participant *i*, and ε*_ij_* ~ *N*(0, σ^2^) is the residual. The main parameters of interest in this analysis are β_CBF_, reflecting the cross-sectional association between CBF and FA or MD; β_time_, reflecting the expectation of annual longitudinal change in FA or MD; and, most important, β_time × CBF_, reflecting the expectation of the difference in the annual change in FA or MD per unit difference in the baseline CBF value. A quadratic age term was not included in the regression model because it did not exhibit statistical significance according to an initial analysis. This was expected, given the limited age range of the participants.

Finally, to investigate whether there was a selection bias in participants between Y25 and Y30, we conducted a complementary and similar analysis restricted to only participants with DTI data obtained at both baseline (Y25) and follow-up (Y30) (eTable 1 in the Supplement) and compared the results with those derived from the main analysis highlighted earlier. The motivation for this sensitivity analysis was to eliminate possible confounding due to participants lost to follow-up. The results of this secondary analysis are presented and discussed in the eAppendix in the Supplement.

In all cases, the threshold for statistical significance was *P* < .05 in 2-sided *t* tests after correction for multiple ROI comparisons using the false discovery rate method.^36^ All statistical calculations were performed with MATLAB version 2021b (MathWorks).

## Results

**Figure 1.**
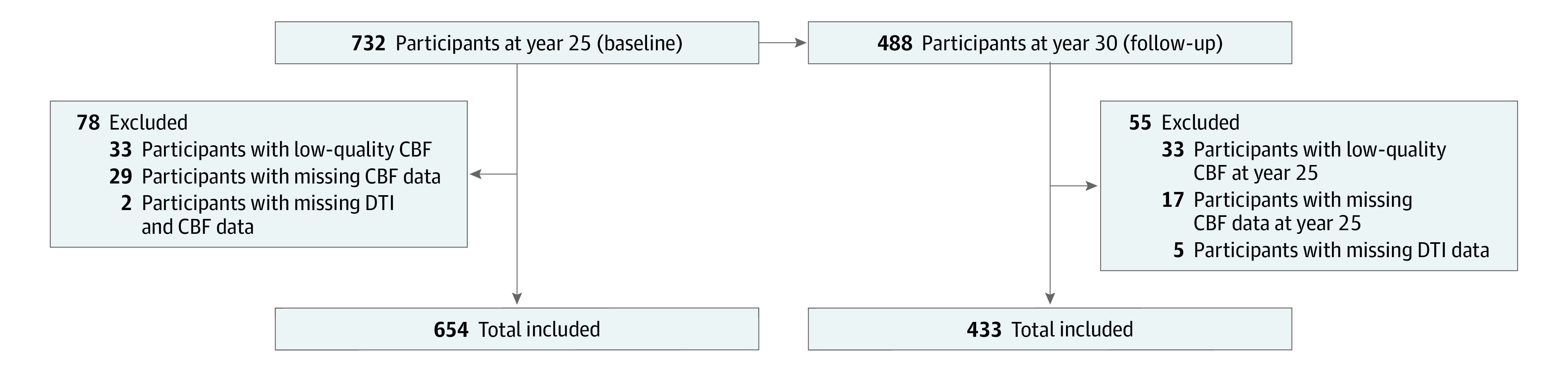
Flowchart Showing Exclusion of Study Participants Cohort demographic characteristics are shown in Table 1. CBF indicates cerebral blood flow; DTI, diffusion tensor imaging.

Table 1 provides demographic characteristics of our final study cohort.^25^ Seventy-eight participants at Y25 were excluded owing to either missing or low-quality ASL or DTI images, whereas 55 participants at Y30 were excluded owing to missing DTI images or missing or low-quality CBF data at Y25 (Figure 1); these participants were excluded from our subsequent statistical analyses. Age did not differ significantly between men and women. The final cohort consisted of 654 participants at baseline (312 men [47.7%] and 342 women [52.3%]; mean [SD] age, 50.3 [3.5] years) and 433 participants at follow-up (203 men [46.9%] and 230 women [53.1%]; mean [SD] age, 55.1 [3.5] years). In the baseline cohort, 247 participants were Black (37.8%), and 394 were White (60.2%); in the follow-up cohort, 156 were Black (36.0%), and 277 were White (64.0%). Further details about participants demographic characteristics can be found in Launer et al.^25^

**Table 1.** Demographic Characteristics of the Participants^a^

Characteristic	Participants, No. (%)	
	Year 25 (baseline)	Year 30 (follow-up)
Total No. of participants	654	433
Sex		
Male	312 (47.7)	203 (46.9)
Female	342 (52.3)	230 (53.1)
Cohort age, y		
Mean (SD)	50.3 (3.5)	55.1 (3.5)
Range	43-56	47-61
Age, mean (SD), y		
Women	50.2 (3.4)	55.2 (3.4)
Men	50.4 (3.5)	55.0 (3.5)
Race		
Black	247 (37.8)	156 (36.0)
White	394 (60.2)	277 (64.0)

^a^ Age did not differ significantly between men and women at baseline or follow-up. The full characterization of this study cohort is provided in Table 1 of Launer et al.^25^

**Figure 2.**
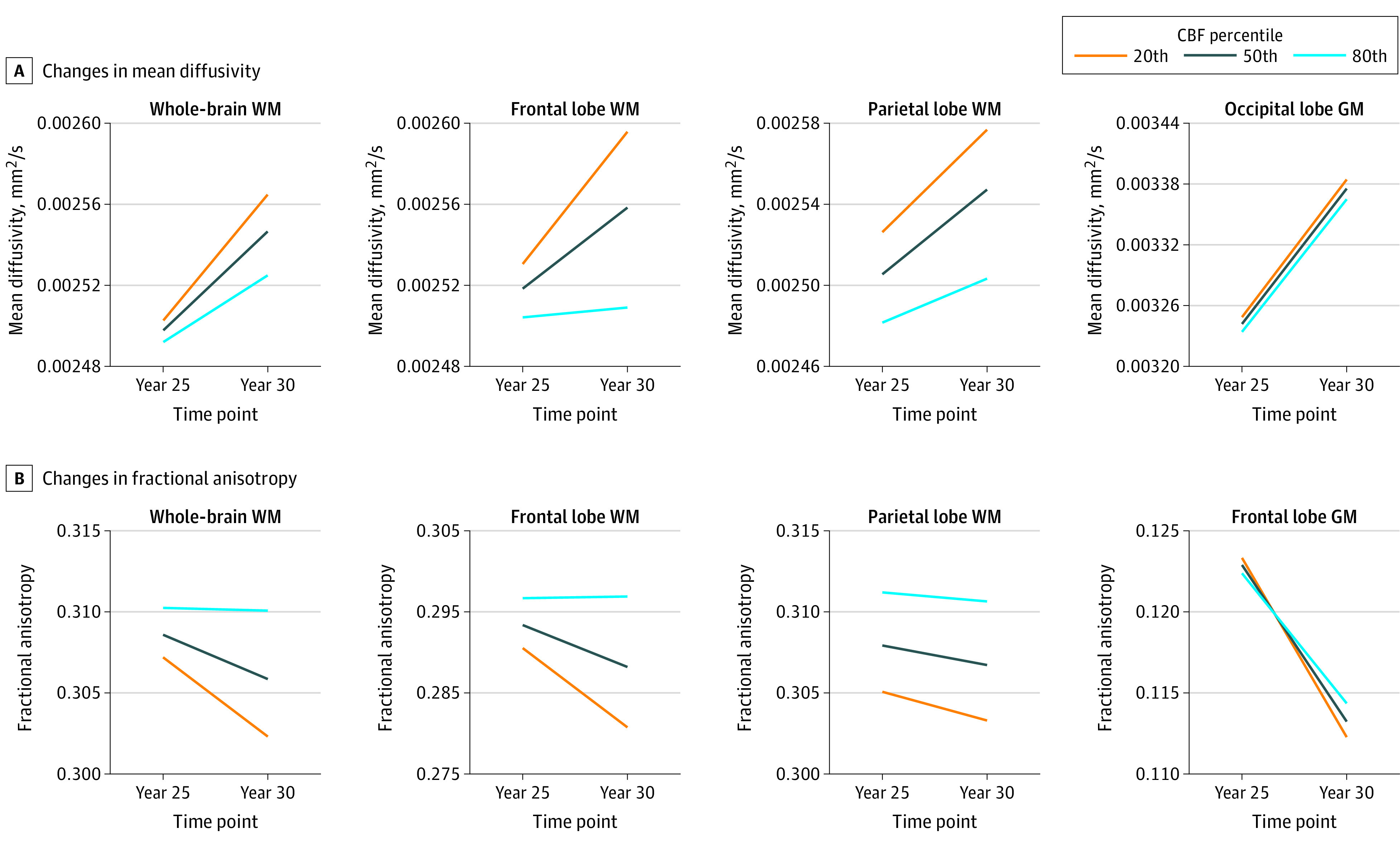
Changes in Mean Diffusivity or Fractional Anisotropy as a Function of Time for 3 Different Values of Cerebral Blood Flow (CBF) Corresponding to the 20th, 50th, and 80th Percentile Values at Baseline Examples are displayed for representative regions of interest. Lower baseline values of CBF flow are associated with steeper deteriorations in cerebral white matter (WM) and gray matter (GM) microstructures (ie, a steeper increase in mean diffusivity or a decrease in fractional anisotropy); this association is less pronounced for higher values of CBF at baseline.

Cross-sectionally, greater CBF was associated with lower MD or higher FA (ie, higher microstructural integrity of GM and WM cerebral tissues) (Table 2 and Table 3). These associations between CBF and FA or MD were statistically significant or close to significant in most ROIs investigated, even after correction for the false discovery rate (Table 2 and Table 3), including the frontal WM lobes (for CBF and MD: mean [SE] β = −1.4 [0.5] × 10^−6^; for CBF and FA: mean [SE] β = 2.9 [1.0] × 10^−4^) and the parietal WM lobes (for CBF and MD: mean [SE] β = −2.4 [0.6] × 10^−6^; for CBF and FA: mean [SE] β = 4.4 [1.1] × 10^−4^). In addition, the steepest slopes in CBF with MD or FA were, overall, found in the anterior regions, including the frontal (for CBF and MD: mean [SE] β = −1.1 [0.6] × 10^−6^; for CBF and FA: mean [SE] β = 2.9 [1.0] × 10^−4^) and parietal lobes (for CBF and MD: mean [SE] β = −1.5 [0.7] × 10^−6^; for CBF and FA: mean [SE] β = 4.4 [1.1] × 10^−4^), whereas the smallest slopes were found in the posterior regions, especially in the occipital lobes (for CBF and MD: mean [SE] β = −0.6 [0.8] × 10^−6^; for CBF and FA: mean [SE] β = 0.4 [0.6] × 10^−4^). Furthermore, and more important, lower CBF values at baseline (Y25) were associated with steeper regional decreases in FA or increases in MD in most ROIs investigated (Figure 2; Table 2 and Table 3). These associations were statistically significant in several brain structures studied (Table 2 and Table 3), including the frontal (for CBF and FA: mean [SE] β = 2.9 [1.0] × 10^−4^) and parietal lobes (for CBF and FA: mean [SE] β = 4.4 [1.1] × 10^−4^). Finally, results derived from analysis restricted to only participants with DTI data obtained at both Y25 and Y30 were consistent with these main observations. The results of this complementary analysis are discussed in detail in the eAppendix in the Supplement (eTables 2 and 3 in the Supplement). Age-, time-, race-, and sex-related differences in DTI indices results are presented and discussed in the eAppendix in the Supplement.

**Table 2.** Regression Coefficient and Significance of the Regression Terms Incorporated in the Linear Mixed-Effects Model^a^

ROI	Age		Sex		Race		CBF		Time		Time × CBF	
	β (SE), ×10^−6^	*P* value	β (SE), ×10^−5^	*P* value	β (SE), ×10^−5^	*P* value	β (SE), ×10^−6^	*P* value	β (SE), ×10^−5^	*P* value	β (SE), ×10^−7^	*P* value
**WB analysis**												
WB												
GM	8.2 (1.9)	<.001	−9.2 (1.4)	<.001	3.1 (1.4)	.052	−0.4 (0.7)	.61	2.7 (0.1)	<.001	−1.2 (1.4)	.45
WM	2.6 (1.2)	.04	−1.2 (0.9)	.21	0.2 (0.9)	.92	−0.7 (0.5)	.47	0.9 (0.1)	<.001	−3.8 (1.1)	.002
FL												
GM	12.1 (2.7)	<.001	−9.2 (2.0)	<.001	6.3 (1.9)	.005	−0.9 (1.0)	.52	2.7 (0.1)	<.001	−1.7 (1.5)	.41
WM	2.8 (1.4)	.06	−0.9 (1.0)	.46	1.6 (1.0)	.20	−1.1 (0.6)	.31	0.7 (0.1)	<.001	−4.4 (1.3)	.002
OL												
GM	5.7 (2.1)	.014	−10.7 (1.6)	<.001	0.4 (1.5)	.92	−0.6 (0.8)	.55	2.9 (0.2)	<.001	0.0 (1.6)	.99
WM	2.0 (1.3)	.13	−2.2 (1.0)	.03	−2.3 (0.9)	.046	−0.6 (0.5)	.47	0.3 (0.1)	.003	−4.1 (1.1)	.002
PL												
GM	11.7 (3.1)	<.001	−14.6 (2.2)	<.001	3.4 (2.2)	.20	−1.3 (1.2)	.47	2.7 (0.2)	<.001	1.8 (1.7)	.42
WM	2.7 (1.6)	.12	−2.7 (1.2)	.03	−0.1 (1.2)	.93	−1.5 (0.7)	.23	0.8 (0.1)	<.001	−4.3 (1.5)	.002
TL												
GM	5.7 (1.6)	<.001	−7.8 (1.1)	<.001	3.7 (1.1)	.005	−0.6 (0.6)	.52	2.8 (0.1)	<.001	−0.3 (1.5)	.91
WM	2.3 (1.1)	.04	−0.2 (0.8)	.77	−0.8 (0.8)	.47	−0.5 (0.4)	.46	1.5 (0.1)	<.001	−2.6 (0.9)	.01
**ROI analysis**												
WB												
GM	8.2 (1.9)	<.001	−9.2 (1.4)	<.001	3.1 (1.4)	.07	−0.4 (0.7)	.87	2.7 (0.1)	<.001	−1.2 (1.4)	.60
WM	2.6 (1.2)	.04	−1.2 (0.9)	.21	0.2 (0.9)	.84	−0.7 (0.5)	.43	0.9 (0.1)	<.001	−3.8 (1.1)	.003
FL												
GM	12.3 (2.7)	<.001	−9.6 (1.9)	<.001	6.5 (1.9)	.003	−0.5 (0.9)	.87	2.7 (0.1)	<.001	−0.6 (1.3)	.83
WM	2.2 (1.4)	.12	−0.3 (1.0)	.77	1.5 (1.0)	.28	−1.4 (0.5)	.008	0.7 (0.1)	<.001	−6.3 (1.1)	<.001
OL												
GM	5.8 (2.1)	.01	−10.6 (1.5)	<.001	0.3 (1.5)	.84	−0.1 (0.7)	.92	2.9 (0.2)	<.001	−2.7 (1.3)	.08
WM	2.5 (1.3)	.07	−3.6 (0.9)	<.001	−1.7 (1.0)	.19	0.4 (0.4)	.65	0.3 (0.1)	.002	−0.1 (0.9)	.95
PL												
GM	10.8 (3.0)	<.001	−11.7 (2.2)	<.001	1.9 (2.2)	.65	−4.4 (1.0)	<.001	2.7 (0.2)	<.001	−0.1 (1.4)	.95
WM	2.6 (1.6)	.11	−2.1 (1.2)	.09	−0.6 (1.2)	.75	−2.4 (0.6)	<.001	0.8 (0.1)	<.001	−2.0 (1.2)	.22
TL												
GM	5.8 (1.6)	<.001	−8.2 (1.1)	<.001	3.9 (1.1)	.003	−0.1 (0.6)	.92	2.8 (0.1)	<.001	−0.8 (1.5)	.83
WM	2.5 (1.1)	.03	−0.7 (0.8)	.40	−0.6 (0.8)	.66	0.1 (0.4)	.92	1.5 (0.1)	<.001	−2.6 (0.9)	.02

Abbreviations: CBF, cerebral blood flow; FL, frontal lobes; GM, gray matter; OL, occipital lobes; PL, parietal lobes; ROI, region of interest; TL, temporal lobes; WB, whole-brain; WM, white matter.

^a^ The mean diffusivity is the dependent variable. Regression coefficient and significance were determined after correction for false discovery rate. The linear mixed-effects model is given by the equation in the Methods.

**Table 3.** Regression Coefficient and Significance of the Regression Terms Incorporated in the Linear Mixed-Effects Model^a^

ROI	Age		Sex		Race		CBF			Time			Time × CBF	
	β (SE), ×10^−4^	*P* value	β (SE), ×10^−3^	*P* value	β (SE), ×10^−3^	*P* value	β (SE), ×10^−4^	*P* value	β (SE), ×10^−3^		*P* value	β (SE), ×10^−5^		*P* value
**WB analysis**														
WB														
GM	−2.5 (1.3)	.16	3.3 (1.0)	.001	−4.4 (0.9)	<.001	−0.0 (0.5)	.98	−2.0 (0.1)		<.001	2.3 (1.2)		.08
WM	−2.9 (2.2)	.27	−4.4 (1.6)	.008	4.8 (1.6)	.003	2.4 (0.9)	.03	−0.5 (0.2)		.01	6.1 (2.0)		.009
FL														
GM	−2.6 (1.4)	.16	3.3 (1.0)	.003	−4.6 (1.0)	<.001	−0.4 (0.6)	.59	−1.9 (0.1)		<.001	2.1 (1.2)		.10
WM	−4.2 (2.4)	.16	−5.1 (1.7)	.005	2.9 (1.7)	.08	2.9 (1.0)	.01	−0.9 (0.2)		<.001	6.5 (1.9)		.007
OL														
GM	−1.6 (1.5)	.31	4.0 (1.1)	<.001	−4.0 (1.1)	<.001	0.4 (0.6)	.58	−2.4 (0.1)		<.001	0.6 (1.4)		.74
WM	−3.1 (2.5)	.28	1.7 (1.8)	.34	5.6 (1.8)	.002	2.1 (1.0)	.10	0.4 (0.2)		.13	6.7 (2.4)		.01
PL														
GM	−3.9 (1.9)	.16	6.5 (1.4)	<.001	−3.8 (1.4)	.006	−0.8 (0.8)	.52	−2.1 (0.1)		<.001	0.1 (1.5)		.97
WM	−1.1 (2.8)	.70	−7.4 (2.1)	<.001	7.4 (2.0)	<.001	4.4 (1.1)	<.001	−0.2 (0.2)		.28	4.9 (2.1)		.04
TL														
GM	−2.5 (1.5)	.16	4.2 (1.1)	<.001	−5.0 (1.1)	<.001	0.4 (0.6)	.59	−2.1 (0.1)		<.001	1.2 (1.4)		.48
WM	−3.7 (2.2)	.16	−2.5 (1.6)	.13	5.9 (1.6)	<.001	1.4 (0.9)	.27	−0.1 (0.2)		.53	6.1 (2.1)		.01
**ROI analysis**														
WB														
GM	−2.5 (1.3)	.14	3.3 (1.0)	.001	−4.4 (0.9)	<.001	−0.0 (0.5)	.98	−2.0 (0.1)		<.001	2.3 (1.2)		.08
WM	−2.9 (2.2)	.25	−4.4 (1.6)	.007	4.8 (1.6)	.004	2.4 (0.9)	.03	−0.5 (0.2)		.01	6.1 (2.0)		.004
FL														
GM	−2.6 (1.4)	.12	3.4 (1.0)	.001	−4.7 (1.0)	<.001	−0.8 (0.5)	.17	−1.9 (0.1)		<.001	3.5 (1.0)		.002
WM	−2.8 (2.3)	.21	−6.4 (1.6)	<.001	3.3 (1.6)	.045	3.7 (0.8)	<.001	−0.9 (0.2)		<.001	11.5 (1.6)		<.001
OL														
GM	−1.9 (1.5)	.25	5.4 (1.1)	<.001	−4.8 (1.1)	<.001	−1.2 (0.5)	.03	−2.4 (0.1)		<.001	0.2 (1.0)		.88
WM	−4.5 (2.5)	.14	5.8 (1.8)	.001	3.8 (1.8)	.04	−0.3 (0.8)	.90	0.4 (0.2)		.14	−7.3 (1.8)		<.001
PL														
GM	−3.6 (1.9)	.14	5.7 (1.4)	<.001	−3.4 (1.4)	.02	0.2 (0.6)	.90	−2.1 (0.1)		<.001	0.8 (1.3)		.59
WM	−1.6 (2.8)	.57	−6.8 (2.0)	.001	7.6 (2.0)	<.001	3.8 (0.9)	<.001	−0.2 (0.2)		.27	1.3 (1.8)		.58
TL														
GM	−2.5 (1.5)	.17	4.1 (1.1)	<.001	−4.9 (1.1)	<.001	0.7 (0.6)	.38	−2.1 (0.1)		<.001	0.8 (1.4)		.59
WM	−4.0 (2.2)	.13	−1.7 (1.6)	.27	5.6 (1.6)	<.001	0.6 (0.9)	.72	−0.1 (0.2)		.53	6.0 (2.1)		.009

Abbreviations: CBF, cerebral blood flow; FL, frontal lobes; GM, gray matter; OL, occipital lobes; PL, parietal lobes; ROI, region of interest; TL, temporal lobes; WB, whole-brain; WM, white matter.

^a^ Fractional anisotropy is the dependent variable. Regression coefficient and significance were determined after correction for false discovery rate. The linear mixed-effects model is given by the equation in the Methods.

## Discussion

Using quantitative MRI of CBF (a measure of blood supply to the brain) and DTI indices of FA and MD (measures of cerebral microstructural tissue integrity), we have demonstrated significant associations between lower CBF at baseline and greater decrease in FA or increase in MD in a large longitudinal cohort of well-characterized adults. Diffusion tensor imaging indices provide quantitative probes of the magnitude and directionality of water molecules. Fractional anisotropy reflects the directionality of molecular displacement by diffusion, with values between 0 and 1; higher values are an indicator of anisotropic diffusion that confers a preferred direction,^17,22,34^ whereas MD reflects the average magnitude of molecular displacement by diffusion, with higher values indicating more freely diffusing water.^17,22^ In WM, decreased FA or increased MD may suggest deterioration of axons or myelin sheaths, whereas in GM, it may suggest loss of synapses or dendrites. Our findings provide evidence of associations between regional variations in blood supply and changes in cerebral tissue status. To our knowledge, this is the first study to demonstrate this association. The results of our observation were obtained from a cohort of middle-aged participants spanning a very limited age range with a relatively restricted follow-up interval of approximately 5 years, which further attests to the strong association between CBF and tissue maintenance, as well as the sensitivity of our MRI metrics to capture these changes.

In agreement with previous work,^8,29^ our results indicate a significant association between CBF and DTI indices, including the frontal WM lobes (for CBF and MD: mean [SE] β = −1.4 [0.5] × 10^−6^; for CBF and FA: mean [SE] β = 2.9 [1.0] × 10^−4^) and the parietal WM lobes (for CBF and MD: mean [SE] β = −2.4 [0.6] × 10^−6^; for CBF and FA: mean [SE] β = 4.4 [1.1] × 10^−4^). Indeed, higher CBF values were significantly associated with lower MD or higher FA values (ie, greater microstructural integrity). In addition, the steepest slopes in CBF with MD or FA were, overall, found in the anterior regions, including the frontal and parietal lobes, whereas the smallest slopes were found in the posterior regions, especially in the occipital lobes. This result supports the retrogenesis paradigm suggesting that early-maturating brain regions (ie, the posterior regions) are spared from neurodegeneration compared with anterior structures.^20,37,38,39,40,41,42,43,44^

The association between lower regional CBF values at baseline and decreased tissue integrity, as reflected by the CBF × time regression term (see the equation in the Methods), was significant in most WM structures investigated. This result agrees with our recent observation of significant regional associations between lower WM CBF and lower myelin content quantified with a specific and direct MRI measure.^32^ Indeed, myelin production or turnover through oligodendrocyte metabolism is an energy-demanding process, making it sensitive to hypoperfusion.^13^ However, this association of CBF with tissue integrity was limited to only a few GM structures. Determination of the DTI indices in GM regions is challenging owing to potential contamination from cerebrospinal fluid. This partial volume effect could bias estimation of FA and MD and precludes accurate evaluation of this association. Indeed, deterioration of GM tissue due to deficits in CBF would be expected because several studies have shown that high consumption of energy is crucial for normal functioning of the brain. It has been shown that a substantial amount of this energy is used to support synaptic activity,^45^ whereas dendritic structures were rapidly distorted after acute or chronic ischemia.^46^ Therefore, inadequate supply of blood glucose or oxygen to the cortical tissue may lead to neuronal and glial death. This paradigm is supported by our results, although significant in limited regions investigated, indicating potential regional associations between lower CBF and lower GM tissue integrity at baseline or increased GM tissue deterioration with time. The use of the free-water DTI technique may provide a more accurate estimation of the DTI indices in GM and a better assessment of the association between CBF and GM status^47,48,49^; this technique represents one of the future directions of our investigations.

As expected, age at baseline was positively associated with MD and negatively associated with FA, indicating lower microstructural integrity at older age (but still middle age) in several brain structures studied. This result agrees with those of several previous investigations.^20,42,50,51,52,53^ Here again, the anterior brain regions, especially the frontal and parietal lobes, exhibited overall the highest slopes, whereas the posterior structures, especially the occipital lobes, exhibited the lowest slopes. This finding agrees with the retrogenesis hypothesis.^20,37,38,39,40,41,42,43,44^ Furthermore, in almost all ROIs investigated, regional increases in MD and decreases in FA (ie, decreased microstructural tissue integrity) were associated with time; this result agrees with results of previous investigations.^54,55,56^ Finally, although women showed significantly lower MD values compared with men overall, and Black participants showed higher MD values compared with White participants overall, these associations were not consistent with FA. A clear interpretation for the associations of sex and race observed in this study is difficult, given the conflicting results across regions for FA and the disagreement between FA and MD. Literature regarding sexual dimorphism and race-related differences in cerebral microstructure remains sparse, requiring further investigations.

### Limitations and Strengths

Our work has limitations. We used identical ASL experimental parameters for all participants and acquired CBF images at a single postlabeling delay, which assumed negligible effects associated with the spatial variation in arterial transit time (ie, the time of the arterial bolus to transit from the labeling plane to the imaging volume). Although this is a reasonable assumption,^24,57^ arterial transit time may vary spatially and may differ between participants owing to arterial blood velocity differences; this could introduce a small bias in derived CBF values. A multiple postlabeling delay ASL technique may provide a more accurate determination CBF.^57^ Moreover, certain physiologic and experimental parameters could bias the determination of the DTI indices. As discussed earlier, partial volume effects from CSF may bias estimation of FA and MD in GM. Furthermore, DTI indices, although sensitive to cerebral microstructural changes, are not specific. Indeed, multiple factors can affect the DTI-derived eigenvalues from which FA and MD are derived; these include myelin breakdown, axonal degeneration, hydration, temperature, flow, macromolecular content, and architectural features, including fiber fanning or crossing. Moreover, the radial diffusivity and axial diffusivity were not analyzed; these DTI metrics have been shown to be more sensitive to myelin content and axonal density, respectively. Unfortunately, these metrics were not available for this study. Finally, our work was conducted on a normatively aging cohort, with future work needed to investigate the potential significance and extent of decreased CBF in the development of cognitive impairment, including dementia. However, the use of advanced MRI metrics in a large and well-characterized cohort of adult participants is an important strength of our study.

## Conclusions

In conclusion, we showed in this longitudinal study that CBF may be significantly associated with cerebral tissue integrity. Further work is needed to shed light on the nature of brain damage in neurodegeneration. These studies may lead to new neuroimaging biomarkers of brain microstructure and function for disease progression.

## Notes

Supplement. **eMethods.** Supplemental Methods **eAppendix.** Supplemental Results **eTable 1. **Participant Demographics for the Supplementary Analysis **eTable 2. **Regression Coefficient (SE) and Significance, After FDR Correction, of the Regression Terms Incorporated in the Linear Mixed-Effects Model Given by Equation 1 **eTable 3. **Regression Coefficient (SE) and Significance, After FDR Correction, of the Regression Terms Incorporated in the Linear Mixed-Effects Model Given by Equation 1
eReferences.

## References

[1] Roher AE, Debbins JP, Malek-Ahmadi M, . Cerebral blood flow in Alzheimer’s disease. Vasc Health Risk Manag. 2012;8:599-611. doi:10.2147/VHRM.S34874 23109807 PMC3481957

[2] Sweeney MD, Kisler K, Montagne A, Toga AW, Zlokovic BV. The role of brain vasculature in neurodegenerative disorders. Nat Neurosci. 2018;21(10):1318-1331. doi:10.1038/s41593-018-0234-x 30250261 PMC6198802

[3] Nasrabady SE, Rizvi B, Goldman JE, Brickman AM. White matter changes in Alzheimer’s disease: a focus on myelin and oligodendrocytes. Acta Neuropathol Commun. 2018;6(1):22. doi:10.1186/s40478-018-0515-3 29499767 PMC5834839

[4] Moll NM, Rietsch AM, Thomas S, . Multiple sclerosis normal-appearing white matter: pathology-imaging correlations. Ann Neurol. 2011;70(5):764-773. doi:10.1002/ana.22521 22162059 PMC3241216

[5] Minkova L, Habich A, Peter J, Kaller CP, Eickhoff SB, Klöppel S. Gray matter asymmetries in aging and neurodegeneration: a review and meta-analysis. Hum Brain Mapp. 2017;38(12):5890-5904. doi:10.1002/hbm.23772 28856766 PMC6866813

[6] Shi Y, Thrippleton MJ, Makin SD, . Cerebral blood flow in small vessel disease: a systematic review and meta-analysis. J Cereb Blood Flow Metab. 2016;36(10):1653-1667. doi:10.1177/0271678X16662891 27496552 PMC5076792

[7] Paolini Paoletti F, Simoni S, Parnetti L, Gaetani L. The contribution of small vessel disease to neurodegeneration: focus on Alzheimer’s disease, Parkinson’s disease and multiple sclerosis. Int J Mol Sci. 2021;22(9):4958. doi:10.3390/ijms22094958 34066951 PMC8125719

[8] Chen JJ, Rosas HD, Salat DH. The relationship between cortical blood flow and sub-cortical white-matter health across the adult age span. PLoS One. 2013;8(2):e56733. doi:10.1371/journal.pone.0056733 23437228 PMC3578934

[9] Brickman AM, Zahra A, Muraskin J, . Reduction in cerebral blood flow in areas appearing as white matter hyperintensities on magnetic resonance imaging. Psychiatry Res. 2009;172(2):117-120. doi:10.1016/j.pscychresns.2008.11.006 19324534 PMC2763417

[10] Brown WR, Moody DM, Thore CR, Challa VR, Anstrom JA. Vascular dementia in leukoaraiosis may be a consequence of capillary loss not only in the lesions, but in normal-appearing white matter and cortex as well. J Neurol Sci. 2007;257(1-2):62-66. doi:10.1016/j.jns.2007.01.015 17320909 PMC1989116

[11] Choi BR, Kim DH, Back DB, . Characterization of white matter injury in a rat model of chronic cerebral hypoperfusion. Stroke. 2016;47(2):542-547. doi:10.1161/STROKEAHA.115.011679 26670084

[12] Cechetti F, Pagnussat AS, Worm PV, . Chronic brain hypoperfusion causes early glial activation and neuronal death, and subsequent long-term memory impairment. Brain Res Bull. 2012;87(1):109-116. doi:10.1016/j.brainresbull.2011.10.006 22040859

[13] Venkat P, Chopp M, Chen J. New insights into coupling and uncoupling of cerebral blood flow and metabolism in the brain. Croat Med J. 2016;57(3):223-228. doi:10.3325/cmj.2016.57.223 27374823 PMC4937223

[14] Kimura N, Nakama H, Nakamura K, Aso Y, Kumamoto T. Effect of white matter lesions on brain perfusion in Alzheimer’s disease. Dement Geriatr Cogn Disord. 2012;34(3-4):256-261. doi:10.1159/000345184 23183589

[15] Pelizzari L, Laganà MM, Di Tella S, . Combined assessment of diffusion parameters and cerebral blood flow within basal ganglia in early Parkinson’s disease. Front Aging Neurosci. 2019;11(134):134. doi:10.3389/fnagi.2019.00134 31214017 PMC6558180

[16] Zhong G, Zhang R, Jiaerken Y, . Better correlation of cognitive function to white matter integrity than to blood supply in subjects with leukoaraiosis. Front Aging Neurosci. 2017;9:185. doi:10.3389/fnagi.2017.00185 28659787 PMC5466957

[17] Basser PJ, Jones DK. Diffusion-tensor MRI: theory, experimental design and data analysis—a technical review. NMR Biomed. 2002;15(7-8):456-467. doi:10.1002/nbm.783 12489095

[18] Alexander AL, Lee JE, Lazar M, Field AS. Diffusion tensor imaging of the brain. Neurotherapeutics. 2007;4(3):316-329. doi:10.1016/j.nurt.2007.05.011 17599699 PMC2041910

[19] Mori S, Zhang J. Principles of diffusion tensor imaging and its applications to basic neuroscience research. Neuron. 2006;51(5):527-539. doi:10.1016/j.neuron.2006.08.012 16950152

[20] Kiely M, Triebswetter C, Cortina LE, . Insights into human cerebral white matter maturation and degeneration across the adult lifespan. Neuroimage. 2022;247:118727. doi:10.1016/j.neuroimage.2021.118727 34813969 PMC8792239

[21] Song S-K, Yoshino J, Le TQ, . Demyelination increases radial diffusivity in corpus callosum of mouse brain. Neuroimage. 2005;26(1):132-140. doi:10.1016/j.neuroimage.2005.01.028 15862213

[22] Pierpaoli C, Barnett A, Pajevic S, . Water diffusion changes in Wallerian degeneration and their dependence on white matter architecture. Neuroimage. 2001;13(6 pt 1):1174-1185. doi:10.1006/nimg.2001.0765 11352623

[23] Detre JA, Leigh JS, Williams DS, Koretsky AP. Perfusion imaging. Magn Reson Med. 1992;23(1):37-45. doi:10.1002/mrm.1910230106 1734182

[24] Alsop DC, Detre JA, Golay X, . Recommended implementation of arterial spin–labeled perfusion MRI for clinical applications: a consensus of the ISMRM perfusion study group and the European consortium for ASL in dementia. Magn Reson Med. 2015;73(1):102-116. doi:10.1002/mrm.25197 24715426 PMC4190138

[25] Launer LJ, Lewis CE, Schreiner PJ, . Vascular factors and multiple measures of early brain health: CARDIA brain MRI study. PLoS One. 2015;10(3):e0122138. doi:10.1371/journal.pone.0122138 25812012 PMC4374951

[26] Alisch JSR, Khattar N, Kim RW, . Sex and age-related differences in cerebral blood flow investigated using pseudo-continuous arterial spin labeling magnetic resonance imaging. Aging (Albany NY). 2021;13(4):4911-4925. doi:10.18632/aging.202673 33596183 PMC7950235

[27] Alsop DC, Dai W, Grossman M, Detre JA. Arterial spin labeling blood flow MRI: its role in the early characterization of Alzheimer’s disease. J Alzheimers Dis. 2010;20(3):871-880. doi:10.3233/JAD-2010-091699 20413865 PMC3643892

[28] Wolk DA, Detre JA. Arterial spin labeling MRI: an emerging biomarker for Alzheimer’s disease and other neurodegenerative conditions. Curr Opin Neurol. 2012;25(4):421-428. doi:10.1097/WCO.0b013e328354ff0a 22610458 PMC3642866

[29] Giezendanner S, Fisler MS, Soravia LM, . Microstructure and cerebral blood flow within white matter of the human brain: a TBSS analysis. PLoS One. 2016;11(3):e0150657. doi:10.1371/journal.pone.0150657 26942763 PMC4778945

[30] Bouhrara M, Spencer RG. Improved determination of the myelin water fraction in human brain using magnetic resonance imaging through bayesian analysis of mcDESPOT. Neuroimage. 2016;127:456-471. doi:10.1016/j.neuroimage.2015.10.034 26499810 PMC4854306

[31] Bouhrara M, Spencer RG. Rapid simultaneous high-resolution mapping of myelin water fraction and relaxation times in human brain using BMC-mcDESPOT. Neuroimage. 2017;147:800-811. doi:10.1016/j.neuroimage.2016.09.064 27729276 PMC5303643

[32] Bouhrara M, Alisch JSR, Khattar N, . Association of cerebral blood flow with myelin content in cognitively unimpaired adults. BMJ Neurol Open. 2020;2(1):e000053. doi:10.1136/bmjno-2020-000053 33681786 PMC7903181

[33] Friedman GD, Cutter GR, Donahue RP, . CARDIA: study design, recruitment, and some characteristics of the examined subjects. J Clin Epidemiol. 1988;41(11):1105-1116. doi:10.1016/0895-4356(88)90080-7 3204420

[34] Le Bihan D, Mangin JF, Poupon C, . Diffusion tensor imaging: concepts and applications. J Magn Reson Imaging. 2001;13(4):534-546. doi:10.1002/jmri.1076 11276097

[35] Morrell CH, Brant LJ, Ferrucci L. Model choice can obscure results in longitudinal studies. J Gerontol A Biol Sci Med Sci. 2009;64(2):215-222. doi:10.1093/gerona/gln024 19196902 PMC2655028

[36] Benjamini Y. Discovering the false discovery rate. J R Stat Soc Series B Stat Methodol. 2010;72(4):405-416. doi:10.1111/j.1467-9868.2010.00746.x

[37] Brickman AM, Meier IB, Korgaonkar MS, . Testing the white matter retrogenesis hypothesis of cognitive aging. Neurobiol Aging. 2012;33(8):1699-1715. doi:10.1016/j.neurobiolaging.2011.06.001 21783280 PMC3222729

[38] Gao J, Cheung RT, Lee TM, . Possible retrogenesis observed with fiber tracking: an anteroposterior pattern of white matter disintegrity in normal aging and Alzheimer’s disease. J Alzheimers Dis. 2011;26(1):47-58. doi:10.3233/JAD-2011-101788 21558648

[39] Reisberg B, Franssen EH, Souren LE, Auer SR, Akram I, Kenowsky S. Evidence and mechanisms of retrogenesis in Alzheimer’s and other dementias: management and treatment import. Am J Alzheimers Dis Other Demen. 2002;17(4):202-212. doi:10.1177/153331750201700411 12184509 PMC10833976

[40] Stricker NH, Schweinsburg BC, Delano-Wood L, . Decreased white matter integrity in late-myelinating fiber pathways in Alzheimer’s disease supports retrogenesis. Neuroimage. 2009;45(1):10-16. doi:10.1016/j.neuroimage.2008.11.027 19100839 PMC2782417

[41] Arshad M, Stanley JA, Raz N. Adult age differences in subcortical myelin content are consistent with protracted myelination and unrelated to diffusion tensor imaging indices. Neuroimage. 2016;143:26-39. doi:10.1016/j.neuroimage.2016.08.047 27561713 PMC5124541

[42] Bouhrara M, Kim RW, Khattar N, . Age-related estimates of aggregate g-ratio of white matter structures assessed using quantitative magnetic resonance neuroimaging. Hum Brain Mapp. 2021;42(8):2362-2373. doi:10.1002/hbm.25372 33595168 PMC8090765

[43] Bouhrara M, Rejimon AC, Cortina LE, . Adult brain aging investigated using BMC-mcDESPOT–based myelin water fraction imaging. Neurobiol Aging. 2020;85:131-139. doi:10.1016/j.neurobiolaging.2019.10.003 31735379 PMC6924176

[44] Yeatman JD, Wandell BA, Mezer AA. Lifespan maturation and degeneration of human brain white matter. Nat Commun. 2014;5:4932. doi:10.1038/ncomms5932 25230200 PMC4238904

[45] Attwell D, Buchan AM, Charpak S, Lauritzen M, Macvicar BA, Newman EA. Glial and neuronal control of brain blood flow. Nature. 2010;468(7321):232-243. doi:10.1038/nature09613 21068832 PMC3206737

[46] Zhu L, Wang L, Ju F, Ran Y, Wang C, Zhang S. Transient global cerebral ischemia induces rapid and sustained reorganization of synaptic structures. J Cereb Blood Flow Metab. 2017;37(8):2756-2767. doi:10.1177/0271678X16674736 27798269 PMC5536786

[47] Golub M, Neto Henriques R, Gouveia Nunes R. Free-water DTI estimates from single b-value data might seem plausible but must be interpreted with care. Magn Reson Med. 2021;85(5):2537-2551. doi:10.1002/mrm.28599 33270935

[48] Pasternak O, Sochen N, Gur Y, Intrator N, Assaf Y. Free water elimination and mapping from diffusion MRI. Magn Reson Med. 2009;62(3):717-730. doi:10.1002/mrm.22055 19623619

[49] Tristán-Vega A, París G, de Luis-García R, Aja-Fernández S. Accurate free-water estimation in white matter from fast diffusion MRI acquisitions using the spherical means technique. Magn Reson Med. 2022;87(2):1028-1035. doi:10.1002/mrm.28997 34463395

[50] Westlye LT, Walhovd KB, Dale AM, . Life-span changes of the human brain white matter: diffusion tensor imaging (DTI) and volumetry. Cereb Cortex. 2010;20(9):2055-2068. doi:10.1093/cercor/bhp280 20032062

[51] Kodiweera C, Alexander AL, Harezlak J, McAllister TW, Wu YC. Age effects and sex differences in human brain white matter of young to middle-aged adults: a DTI, NODDI, and q-space study. Neuroimage. 2016;128:180-192. doi:10.1016/j.neuroimage.2015.12.033 26724777 PMC4824064

[52] Westlye LT, Walhovd KB, Dale AM, . Life-span changes of the human brain white matter: diffusion tensor imaging (DTI) and volumetry. Cereb Cortex. 2010;20(9):2055-2068. doi:10.1093/cercor/bhp280 20032062

[53] Qian W, Khattar N, Cortina LE, Spencer RG, Bouhrara M. Nonlinear associations of neurite density and myelin content with age revealed using multicomponent diffusion and relaxometry magnetic resonance imaging. Neuroimage. 2020;223:117369. doi:10.1016/j.neuroimage.2020.117369 32931942 PMC7775614

[54] Sullivan EV, Rohlfing T, Pfefferbaum A. Longitudinal study of callosal microstructure in the normal adult aging brain using quantitative DTI fiber tracking. Dev Neuropsychol. 2010;35(3):233-256. doi:10.1080/87565641003689556 20446131 PMC2867078

[55] de Groot M, Cremers LG, Ikram MA, . White matter degeneration with aging: longitudinal diffusion MR imaging analysis. Radiology. 2016;279(2):532-541. doi:10.1148/radiol.2015150103 26536311

[56] Beck D, de Lange AG, Maximov II, . White matter microstructure across the adult lifespan: a mixed longitudinal and cross-sectional study using advanced diffusion models and brain-age prediction. Neuroimage. 2021;224:117441. doi:10.1016/j.neuroimage.2020.117441 33039618

[57] Qin Q, Huang AJ, Hua J, Desmond JE, Stevens RD, van Zijl PCM. Three-dimensional whole-brain perfusion quantification using pseudo-continuous arterial spin labeling MRI at multiple post-labeling delays: accounting for both arterial transit time and impulse response function. NMR Biomed. 2014;27(2):116-128. doi:10.1002/nbm.3040 24307572 PMC3947417

