# Supplementary material for: Association of Cerebral Blood Flow With Longitudinal Changes in Cerebral Microstructural Integrity in the Coronary Artery Risk Development in Young Adults (CARDIA) Study
Source: JAMA Netw Open. 2022 Sep 12;5(9):e2231189. doi: 10.1001/jamanetworkopen.2022.31189 (PMC9468885; doi:10.1001/jamanetworkopen.2022.31189)
Supplement: Supplement. — eMethods. Supplemental Methods eAppendix. Supplemental Results eTable 1. Participant Demographics for the Supplementary Analysis eTable 2. Regression Coefficient (SE) and Significance, After FDR Correction, of the Regression Terms Incorporated in the Linear Mixed-Effects Model Given by Equation 1 eTable 3. Regression Coefficient (SE) and Significance, After FDR Correction, of the Regression Terms Incorporated in the Linear Mixed-Effects Model Given by Equation 1 eReferences. [file jamanetwopen-e2231189-s001.pdf]

Supplementary Online Content

Bouhrara M, Triebswetter C, Kiely M, et al. Association of cerebral blood flow with longitudinal changes in cerebral microstructural integrity in the Coronary Artery Risk Development in Young Adults (CARDIA) study. *JAMA Netw Open*. 2022;5(9):e2231189. doi:10.1001/jamanetworkopen.2022.31189

eMethods. Supplemental Methods

eAppendix. Supplemental Results

eTable 1. Participant Demographics for the Supplementary Analysis

eTable 2. Regression Coefficient (SE) and Significance, After FDR Correction, of the Regression Terms Incorporated in the Linear Mixed-Effects Model Given by Equation 1

eTable 3. Regression Coefficient (SE) and Significance, After FDR Correction, of the Regression Terms Incorporated in the Linear Mixed-Effects Model Given by Equation 1

eReferences

This supplementary material has been provided by the authors to give readers additional information about their work.

© 2022 Bouhrara M et al. *JAMA Network Open.*

eMethods. Supplemental Methods

Study cohort

Participants data were drawn from the Coronary Artery Risk Development in Young Adults (CARDIA) study. Established in 1985, CARDIA is a longitudinal clinical study of development and cardiovascular disease determinants in adults. Participants were recruited from four United States cities, namely, Birmingham, Alabama; Chicago, Illinois; Minneapolis, Minnesota; and Oakland, California. All participants provided written informed consent at each time of examination, and the study was approved, annually, by the institutional review boards from all coordinating centers, namely, the University of Alabama Birmingham (UAB) Institutional Review Board, the University of Minnesota Institutional (UMI) Review Board, and the Kaiser Permanente Northern California Institutional (NCI) Review Board. Further details about the CARDIA study can be found in (1). In this study, we used the MRI data of cerebral blood flow (CBF) acquired at 25 years (Y25) of follow-up, which represent the first timepoint of CBF MRI acquisition in the CARDIA study, and of the diffusion tensor imaging (DTI) MRI data acquired at 25 years (Y25) and 30 years (Y30) of follow-up. This study followed the Strengthening the Reporting of Observational Studies in Epidemiology (STROBE) reporting guideline.

MRI data acquisition

Whole-brain MR images were acquired on three 3T MR scanners located proximal to each CARDIA clinic site (NCI: Siemens 3T Tim Trio/VB 15 platform; UMI: Siemens 3T Tim Trio/VB 15 platform, and UAB: Philips 3T Achieva/2.6.3.6 platform). We note that all brain MRI scans were performed per CARDIA protocol using 3 T MRI machines, and standardized across machines using a common machine head phantom (Oakland: Siemens [Munich, Germany] 3T Tim Trio/VB 15 platform; Minneapolis: Siemens 3T Tim Trio/VB 15 platform; and Birmingham: Philips [Best, the Netherlands] 3T Achieva/2.6.3.6 platform) (1).

Image processing, parameter mapping, and regions-of-interest selection

Image processing was performed by the Biomedical Image Analysis Section of Department of Radiology at University of Pennsylvania. Briefly, an initial quality control (QC) protocol was conducted to identify motion artifacts or other quality issues. Images failing this test were flagged for further inspection and were subsequently discarded from our analysis. QC checks were

© 2022 Bouhrara M et al. *JAMA Network Open.*

performed on intermediate and final processing steps by visual inspection and by identifying outliers of calculated variable or parameter distributions. Following the QC protocol, the pseudo-continuous ASL (pCASL) (2) and DTI (3, 4) MRI datasets were processed using automated pipelines for the calculation of the corresponding CBF or FA and MD maps, respectively. Briefly, the DTI and the ASL derived CBF maps were registered to the subject T_1-weigthed images using FSL, from which the mean values of the different regions of interest were obtained. DTI was registered to the T_1 space using FSL FLIRT, while the ASL images were registered to the T_1-weigthed images using the FSL boundary-based registration. The detailed description of the QC procedure and parameters mapping analyses can be found in (1, 5-8). Ten regions of interest (ROIs) were defined corresponding to whole brain GM or WM, and frontal, parietal, temporal and occipital lobe WM or GM. These regional brain volumes were obtained using T_1-weighted images. The processing steps include reorientation, inhomogeneity correction with N4 (9) and multi-atlas skull-stripping (10). Thereafter, the anatomical regions of interest were segmented using a multi-atlas segmentation method (6). Within each WM or GM ROI, the mean FA and MD values were calculated, while the mean CBF values were calculated in the GM ROIs only. Our main analysis was restricted to GM CBF due to the high sensitivity to noise of CBF values derived from ASL in WM ROIs in addition to the fact that the pCASL protocol used here was optimized for GM CBF determination (2).

© 2022 Bouhrara M et al. *JAMA Network Open.*

eAppendix. Supplemental Results

Age, time, race, and sex-related differences in DTI indices

As expected, older individuals had higher MD and lower FA at baseline, indicating lower microstructural integrity at older ages (Tables. 2 and 3); these associations were statistically significant ($p < 0.05$) or close to significance ($p < 0.1$) in several ROIs studied. Here again, the anterior brain regions, especially the frontal and parietal lobes exhibited, overall, the highest slopes, while the posterior structures, especially the occipital lobes, exhibited the lowest slopes. Furthermore, in almost all ROIs investigated, regional increases in MD or decreases in FA, that is, decreased microstructural tissue integrity, were associated with time (Fig. 2, Tables 2 and 3). Moreover, although women showed, overall, significantly lower MD values as compared to men, and Black participants showed, overall, higher MD values as compared to White participants, these associations were not consistent with FA (Table 2 and 3). Finally, results from analysis restricted to only participants with DTI data obtained at both Y25 and Y30, were consistent with these main observations. The results of this complementary analysis are discussed in detail in the Supplementary Material (eTables 2-3).

Supplementary statistical analysis

Here, we investigated whether there was a selection bias in participants between Y25 and Y30. For that, we conducted a complementary and similar analysis to the main analysis presented in the main document but restricted to only participants with DTI data obtained at both baseline (*i.e.*, Y25) and follow-up (*i.e.*, Y30) (eTable 1). The motivation for this sensitivity analysis was to eliminate possible confounding due to participants lost to follow up. We compared the results to those derived from the main analysis highlighted in the main document.

First, for each ROI, CBF values of participants with 1 (group 1) or 2 (group 2) visits were compared using the two-sample *t*-test and Kolmogorov-Smirnov test. Both tests indicated no significant differences in CBF distributions between the two groups in all ROIs. Furthermore, the results of the regression analysis are virtually identical to those of the main analysis. Indeed, although the statistical significance of the independent parameters on the changes in FA and MD was, overall, lower in this analysis, the regression coefficients of the longitudinal independent parameters, that is, time and time × CBF, were very similar between both analyses (eTables 2-3).

© 2022 Bouhrara M et al. *JAMA Network Open.*

eTable 1. Participant Demographics for the Supplementary Analysis

Age did not differ significantly ($p > 0.1$) between men and women. SD: standard deviation; Min: minimum; Max: maximum; Yrs.: years.

**Characteristic**	**Y25 (baseline)**	**Y30 (follow-up)**
**Number of participants**	433 (230 women)	433 (230 women)
**Mean (SD age (yrs.)**	50.4 (3.4	55.1 (3.5
**Min / max age (yrs.)**	43 / 56	47 / 61

© 2022 Bouhrara M et al. *JAMA Network Open.*

eTable 2. Regression Coefficient (SE) and Significance, After FDR Correction, of the Regression Terms Incorporated in the Linear Mixed-Effects Model Given by Equation 1

Here, the mean diffusivity (MD) is the dependent variable.

WB-analysis

	**Age**		**Sex**		**Race**		**CBF**		**Time**		**Time x CBF**	
**ROI**	β(SE) × 10^{-6}	*p*	β(SE) × 10^{-5}	*p*	β(SE) × 10^{-5}	*p*	β(SE) × 10^{-6}	*p*	β(SE) × 10^{-5}	*p*	β(SE) × 10^{-7}	*p*
**WB GM**	5.2 (2.2)	0.105	-9.8 (1.6)	0.000	2.9 (1.6)	0.162	-0.5 (0.9)	0.613	2.6 (0.1)	0.000	-1.2 (1.4)	0.462
**WB WM**	0.9 (1.4)	0.715	-1.7 (1.0)	0.109	-0.1 (1.0)	0.993	-0.7 (0.6)	0.531	0.9 (0.1)	0.000	-3.8 (1.1)	0.003
**FL GM**	7.3 (3.2)	0.105	-9.1 (2.3)	0.000	6.3 (2.3)	0.060	-1.3 (1.3)	0.531	2.6 (0.1)	0.000	-1.7 (1.5)	0.412
**FL WM**	0.9 (1.7)	0.715	-1.5 (1.2)	0.227	1.3 (1.2)	0.376	-1.0 (0.7)	0.531	0.7 (0.1)	0.000	-4.5 (1.3)	0.003
**OL GM**	3.5 (2.5)	0.304	-11.4 (1.8)	0.000	0.6 (1.8)	0.945	-0.8 (1.0)	0.578	2.9 (0.2)	0.000	0.2 (1.7)	0.902
**OL WM**	0.3 (1.5)	0.835	-2.9 (1.1)	0.016	-2.2 (1.1)	0.153	-0.6 (0.6)	0.530	0.3 (0.1)	0.004	-4.0 (1.2)	0.003
**PL GM**	6.7 (3.7)	0.168	-14.9 (2.7)	0.000	3.4 (2.6)	0.325	-1.7 (1.5)	0.531	2.6 (0.2)	0.000	1.8 (1.7)	0.412
**PL WM**	0.7 (1.9)	0.806	-2.8 (1.4)	0.065	0.0 (1.4)	0.993	-1.6 (0.8)	0.487	0.8 (0.1)	0.000	-4.2 (1.5)	0.013
**TL GM**	3.9 (1.9)	0.115	-9.0 (1.3)	0.000	3.1 (1.3)	0.106	-0.5 (0.8)	0.578	2.8 (0.2)	0.000	-0.3 (1.6)	0.902
**TL WM**	0.7 (1.2)	0.715	-1.3 (0.9)	0.180	-1.3 (0.9)	0.268	-0.4 (0.5)	0.588	1.4 (0.1)	0.000	-2.6 (1.0)	0.015

ROI-analysis

	**Age**		**Sex**		**Race**		**CBF**		**Time**		**Time x CBF**	
**ROI**	β(SE) × 10^{-6}	*p*	β(SE) × 10^{-5}	*p*	β(SE) × 10^{-5}	*p*	β(SE) × 10^{-6}	*p*	β(SE) × 10^{-5}	*p*	β(SE) × 10^{-7}	*p*
**WB GM**	5.2 (2.2)	0.091	-9.8 (1.6)	0.000	2.9 (1.6)	0.216	-0.5 (0.9)	0.703	2.6 (0.1)	0.000	-1.2 (1.4)	0.616
**WB WM**	0.9 (1.4)	0.662	-1.7 (1.0)	0.109	-0.1 (1.0)	0.994	-0.7 (0.6)	0.542	0.9 (0.1)	0.000	-3.8 (1.1)	0.004
**FL GM**	7.6 (3.2)	0.090	-9.9 (2.3)	0.000	6.7 (2.3)	0.034	-0.5 (1.1)	0.703	2.6 (0.1)	0.000	-0.6 (1.3)	0.788
**FL WM**	0.2 (1.6)	0.884	-0.8 (1.2)	0.514	1.1 (1.2)	0.556	-1.4 (0.6)	0.050	0.7 (0.1)	0.000	-6.4 (1.1)	0.000
**OL GM**	3.6 (2.4)	0.292	-10.6 (1.8)	0.000	0.0 (1.8)	0.994	-1.0 (0.8)	0.515	2.9 (0.2)	0.000	-2.1 (1.3)	0.263
**OL WM**	1.0 (1.5)	0.662	-4.4 (1.1)	0.000	-1.5 (1.1)	0.427	0.2 (0.5)	0.700	0.3 (0.1)	0.004	0.1 (0.9)	0.906
**PL GM**	5.2 (3.6)	0.292	-11.2 (2.6)	0.000	1.2 (2.6)	0.809	-5.4 (1.2)	0.000	2.6 (0.2)	0.000	0.2 (1.5)	0.908

© 2022 Bouhrara M et al. *JAMA Network Open.*

**PL WM**	0.5 (1.9)	0.868	-2.2 (1.4)	0.123	-0.6 (1.4)	0.809	-2.6 (0.7)	0.000	0.8 (0.1)	0.000	-1.8 (1.3)	0.310
**TL GM**	4.1 (1.9)	0.091	-9.3 (1.3)	0.000	3.2 (1.3)	0.081	0.0 (0.8)	0.988	2.8 (0.2)	0.000	-0.8 (1.6)	0.788
**TL WM**	1.0 (1.2)	0.662	-1.8 (0.9)	0.057	-1.1 (0.9)	0.427	0.3 (0.5)	0.701	1.4 (0.1)	0.000	-2.7 (1.0)	0.019

CBF: cerebral blood flow, ROI: region-of-interest, WB: whole brain, WM: white matter, GM: gray matter, FL: frontal lobes, OL: occipital lobes, PL: parietal lobes, TL: temporal lobes.

© 2022 Bouhrara M et al. *JAMA Network Open.*

eTable 3. Regression Coefficient (SE) and Significance, After FDR Correction, of the Regression Terms Incorporated in the Linear Mixed-Effects Model Given by Equation 1

Here, the fractional anisotropy (FA) is the dependent variable.

**WB-analysis**												
	**Age**		**Sex**		**Race**		**CBF**		**Time**		**Time x CBF**	
**ROI**	β(SE) × 10^{-4}	*p*	β(SE) × 10^{-3}	*p*	β(SE) × 10^{-3}	*p*	β(SE) × 10^{-4}	*p*	β(SE) × 10^{-3}	*p*	β(SE) × 10^{-5}	*p*
**WB GM**	-2.0 (1.6)	0.570	3.6 (1.2)	0.006	-4.3 (1.2)	0.001	0.3 (0.7)	0.795	-2.0 (0.1)	0.000	2.0 (1.2)	0.186
**WB WM**	-1.6 (2.7)	0.662	-3.0 (1.9)	0.132	4.9 (1.9)	0.015	2.2 (1.1)	0.163	-0.5 (0.2)	0.011	6.1 (2.1)	0.015
**FL GM**	-1.4 (1.7)	0.576	3.1 (1.2)	0.025	-4.4 (1.2)	0.001	0.1 (0.7)	0.872	-1.9 (0.1)	0.000	1.8 (1.2)	0.197
**FL WM**	-2.6 (2.9)	0.570	-3.3 (2.1)	0.132	3.0 (2.1)	0.150	2.9 (1.2)	0.084	-1.0 (0.2)	0.000	6.4 (2.0)	0.015
**OL GM**	-1.4 (1.8)	0.567	4.3 (1.3)	0.003	-4.5 (1.3)	0.001	0.5 (0.8)	0.736	-2.3 (0.1)	0.000	0.4 (1.4)	0.881
**OL WM**	-2.2 (3.0)	0.571	3.4 (2.2)	0.132	5.0 (2.1)	0.025	1.6 (1.3)	0.491	0.3 (0.2)	0.212	6.8 (2.5)	0.016
**PL GM**	-3.1 (2.4)	0.752	6.5 (1.7)	0.001	-2.9 (1.7)	0.100	-0.3 (1.0)	0.872	-2.1 (0.2)	0.000	-0.2 (1.6)	0.881
**PL WM**	0.6 (3.5)	0.872	-6.0 (2.5)	0.027	7.2 (2.5)	0.006	4.1 (1.4)	0.036	-0.2 (0.2)	0.300	4.9 (2.2)	0.046
**TL GM**	-2.1 (1.9)	0.576	4.6 (1.3)	0.003	-4.5 (1.3)	0.001	0.8 (0.8)	0.577	-2.1 (0.1)	0.000	0.8 (1.4)	0.726
**TL WM**	-2.6 (2.6)	0.507	-1.0 (1.9)	0.619	6.5 (1.9)	0.001	0.9 (1.1)	0.721	-0.1 (0.2)	0.523	6.4 (2.2)	0.015

**ROI-analysis**												
	**Age**		**Sex**		**Race**		**CBF**		**Time**		**Time x CBF**	
**ROI**	β(SE) × 10^{-4}	*p*	β(SE) × 10^{-3}	*p*	β(SE) × 10^{-3}	*p*	β(SE) × 10^{-4}	*p*	β(SE) × 10^{-3}	*p*	β(SE) × 10^{-5}	*p*
**WB GM**	-2.0 (1.6)	0.529	3.6 (1.2)	0.005	-4.3 (1.2)	0.000	0.3 (0.7)	0.706	-2.0 (0.1)	0.000	2.0 (1.2)	0.186
**WB WM**	-1.6 (2.7)	0.700	-3.0 (1.9)	0.130	4.9 (1.9)	0.015	2.2 (1.1)	0.163	-0.5 (0.2)	0.011	6.1 (2.1)	0.006
**FL GM**	-1.4 (1.7)	0.564	3.3 (1.2)	0.010	-4.5 (1.2)	0.000	-0.5 (0.6)	0.523	-1.9 (0.1)	0.000	3.3 (1.1)	0.006
**FL WM**	-0.8 (2.8)	0.858	-5.3 (2.0)	0.010	3.6 (2.0)	0.08	4.2 (1.0)	0.000	-0.9 (0.2)	0.000	11.1 (1.7)	0.000
**OL GM**	-1.8 (1.8)	0.529	5.6 (1.3)	0.000	-5.1 (1.3)	0.000	-0.8 (0.6)	0.387	-2.3 (0.1)	0.000	-0.3 (1.1)	0.787
**OL WM**	-3.9 (2.9)	0.529	8.3 (2.1)	0.000	2.5 (2.1)	0.241	-0.8 (1.0)	0.520	0.3 (0.2)	0.232	-7.2 (1.9)	0.000
**PL GM**	-2.6 (2.4)	0.528	5.4 (1.7)	0.003	-2.3 (1.7)	0.191	0.8 (0.8)	0.523	-2.1 (0.2)	0.000	0.5 (1.3)	0.770

© 2022 Bouhrara M et al. *JAMA Network Open.*

**PL WM**	0.0 (3.5)	0.996	-5.1 (2.5)	0.049	7.3 (2.5)	0.006	3.4 (1.2)	0.020	-0.2 (0.2)	0.287	1.4 (1.8)	0.633
**TL GM**	-2.1 (1.8)	0.529	4.6 (1.3)	0.001	-4.4 (1.3)	0.002	1.0 (0.8)	0.387	-2.1 (0.1)	0.000	0.6 (1.4)	0.771
**TL WM**	-3.1 (2.6)	0.524	0.0 (1.9)	0.994	6.2 (1.9)	0.002	-0.2 (1.1)	0.862	-0.1 (0.2)	0.528	6.6 (2.2)	0.006

CBF: cerebral blood flow, ROI: region-of-interest, WB: whole brain, WM: white matter, GM: gray matter, FL: frontal lobes, OL: occipital lobes, PL: parietal lobes, TL: temporal lobes.

© 2022 Bouhrara M et al. *JAMA Network Open.*

eReferences

1. Launer LJ, Lewis CE, Schreiner PJ, Sidney S, Battapady H, Jacobs DR, et al. Vascular factors and multiple measures of early brain health: CARDIA brain MRI study. PloS one. 2015;10(3):e0122138-e.
2. Alsop DC, Detre JA, Golay X, Günther M, Hendrikse J, Hernandez-Garcia L, et al. Recommended Implementation of Arterial Spin Labeled Perfusion MRI for Clinical Applications: A consensus of the ISMRM Perfusion Study Group and the European Consortium for ASL in Dementia. Magnetic resonance in medicine. 2015;73(1):102-16.
3. Basser PJ, Jones DK. Diffusion-tensor MRI: theory, experimental design and data analysis - a technical review. NMR in biomedicine. 2002;15(7-8):456-67.
4. Le Bihan D, Mangin JF, Poupon C, Clark CA, Pappata S, Molko N, et al. Diffusion tensor imaging: concepts and applications. Journal of magnetic resonance imaging : JMRI. 2001;13(4):534-46.
5. Goldszal AF, Davatzikos C, Pham DL, Yan MX, Bryan RN, Resnick SM. An image-processing system for qualitative and quantitative volumetric analysis of brain images. Journal of computer assisted tomography. 1998;22(5):827-37.
6. Doshi J, Erus G, Ou Y, Resnick SM, Gur RC, Gur RE, et al. MUSE: MUlti-atlas region Segmentation utilizing Ensembles of registration algorithms and parameters, and locally optimal atlas selection. NeuroImage. 2016;127:186-95.
7. Dolui S, Tisdall D, Vidorreta M, Jacobs DR, Nasrallah IM, Bryan RN, et al. Characterizing a perfusion-based periventricular small vessel region of interest. NeuroImage: Clinical. 2019;23:101897.
8. Dolui S, Wang Z, Wang DJ, Mattay R, Finkel M, Elliott M, et al. Comparison of non-invasive MRI measurements of cerebral blood flow in a large multisite cohort. Journal of Cerebral Blood Flow & Metabolism. 2016;36(7):1244-56.
9. Tustison NJ, Avants BB, Cook PA, Zheng Y, Egan A, Yushkevich PA, et al. N4ITK: improved N3 bias correction. IEEE transactions on medical imaging. 2010;29(6):1310-20.
10. Doshi J, Erus G, Ou Y, Gaonkar B, Davatzikos C. Multi-atlas skull-stripping. Acad Radiol. 2013;20(12):1566-76.

© 2022 Bouhrara M et al. *JAMA Network Open.*
